# Human Capital and Administrative Burden: The Role of Cognitive Resources in Citizen‐State Interactions

**DOI:** 10.1111/puar.13134

**Published:** 2019-12-15

**Authors:** Julian Christensen, Lene Aarøe, Martin Baekgaard, Pamela Herd, Donald P. Moynihan

**Affiliations:** ^1^ Aarhus University; ^2^ Georgetown University

## Abstract

*One means by which the state reinforces inequality is by imposing administrative burdens that loom larger for citizens with lower levels of human capital. Integrating insights from various disciplines, this article focuses on one aspect of human capital: cognitive resources. The authors outline a model that explains how burdens and cognitive resources, especially executive functioning, interrelate. The article then presents illustrative examples, highlighting three common life factors—scarcity, health problems, and age‐related cognitive decline. These factors create a human capital catch‐22, increasing people*'*s likelihood of needing state assistance while simultaneously undermining the cognitive resources required to negotiate the burdens they encounter while seeking such assistance. The result is to reduce access to state benefits and increase inequality. The article concludes by calling for scholars of behavioral public administration and public administration more generally to incorporate more attention to human capital into their research*.

**Evidence for Practice**

- People's human capital influences how they engage with administrative processes. Groups with lower levels of human capital struggle more with administrative burdens; therefore, they are less likely to access public services.
- A citizen‐centered approach to public administration implies that policy makers and administrators take account of variation in human capital when making choices about policy design and implementation.
- Cognitive resources, including executive functioning, are a form of human capital, which is key to citizens' ability to initiate and master state interactions.
- Citizens experiencing scarcity, health problems, and cognitive decline face a catch‐22: such common life factors make them more likely to need to engage with the state, but reduce their executive functioning, making them less able to deal with administrative burdens.

The idea of administrative burden is intuitive. We recognize it from our personal experiences of engaging with the state. It reflects the time we spend getting a new driver's license or the lines we stand in when we vote. For many, it also reflects the difficulty of accessing public services such as income supports or health care. All settings in which citizens engage with the state are, to varying degrees, venues where actions by the state affect the learning, compliance, and psychological costs that citizens encounter.

Administrative burdens—and the related concept of “sludge”—deserve attention for a number of reasons (Herd and Moynihan 2018; Sunstein 2019a; Thaler 2018). First, they can have significant effects on citizen outcomes, such as whether individuals can access the benefits they need and the rights they are entitled to (e.g., Heinrich 2018) or whether they engage in civic and electoral participation (Bruch, Ferree, and Soss 2010). Linos and Riesch (2019) even find that administrative burdens in recruitment processes affect people's access to public sector jobs.

Second, burdens have distributive effects: they hurt some groups more than others. Some individuals encounter burdens more frequently because of the kinds of programs they seek to access. Means‐tested programs will, because of their inherent need to verify eligibility, be more burdensome than a universal program designed to be accessible to nearly everyone (Soss 1999). Furthermore, some people may simply be better able to deal with burdensome state actions because they have higher levels of human capital. By human capital, we mean the stock of innate abilities and characteristics that people possess and the knowledge and skills they acquire over time. Economists traditionally define human capital as the characteristics that contribute to firm profitability and higher compensation (e.g., Becker 1962), but the concept also has applications beyond economic situations. Human capital can include knowledge, personality traits, health, experiences, education, and cognitive functioning (Becker 1993; Grossman 1972; Laroche, Mérette, and Ruggeri 1999). In practice, those with low human capital, such as low education or poor mental or physical health, will be more likely to rely on means‐tested programs.

In this article, we discuss how factors that increase people's need of assistance from the state also decrease their ability to cope with burdensome state actions. Our primary contribution is to identify some micro‐foundations that improve on existing explanations of variations in people's experiences of administrative burdens and negative distributive outcomes resulting from these variations. In doing so, we theorize about the effects of human capital, particularly cognitive resources, on people's experience of administrative burdens.

We contribute a novel approach to understanding inequalities arising from citizen‐state interactions. The limited attention that the field of public administration has given to issues of equity is typically framed in terms of social equity for different sociodemographic groups (Frederickson 2015). A consideration of human capital offers a related but distinct perspective, one that draws on evidence from behavioral science, psychology, and health. The administrative burden framework has a distinctly behavioral focus—it focuses on how people experience and respond to stimuli. Our approach enriches this framework and behavioral public administration more broadly by considering underlying human capital differences that help explain both standard behavioral factors that constrain rationality (e.g., Battaglio et al. 2019) as well as outcomes such as program take‐up.

To develop our framework, it is necessary to first distinguish between state actions, on the one hand, and citizens' experience of administrative burden, on the other, which we do in the next section. We then outline ways in which cognitive resources (particularly executive functioning) influence people's experiences of burden. To illustrate the importance of these theoretical arguments, we next discuss how common life factors (scarcity, health problems, and age‐related cognitive decline) both increase people's likelihood of needing state assistance and undermine their cognitive resources, alerting us to a human capital catch‐22 in citizen‐state interactions. We conclude by discussing the policy implications of these insights.

## Distinguishing between State Actions and Experiences of Burdens

A simple definition of administrative burden is that it is an individual's experience of a policy's implementation as onerous (Burden et al. 2012). A more comprehensive definition frames administrative burden as the experience of three types of costs in public settings (Herd and Moynihan 2018). First, learning costs arise when people seek information about the existence and eligibility criteria of programs, public goods, or individual rights. Second, compliance costs arise from the time, effort, and financial costs of meeting administrative demands. Third, psychological costs arise in the form of stigma from participating in unpopular programs, the experience of disempowerment, feelings of subservience and loss of autonomy, and related stress (e.g., see Hattke, Hensel, and Kalucza in this symposium on negative emotional reactions as psychological costs). The presence and effects of the costs in any particular setting is an empirical question, since costs may vary across policies and individuals. In some settings, psychological costs, for instance, may be very relevant while learning, and compliance costs may be low. For others, all costs may be salient.

**Figure 1:**
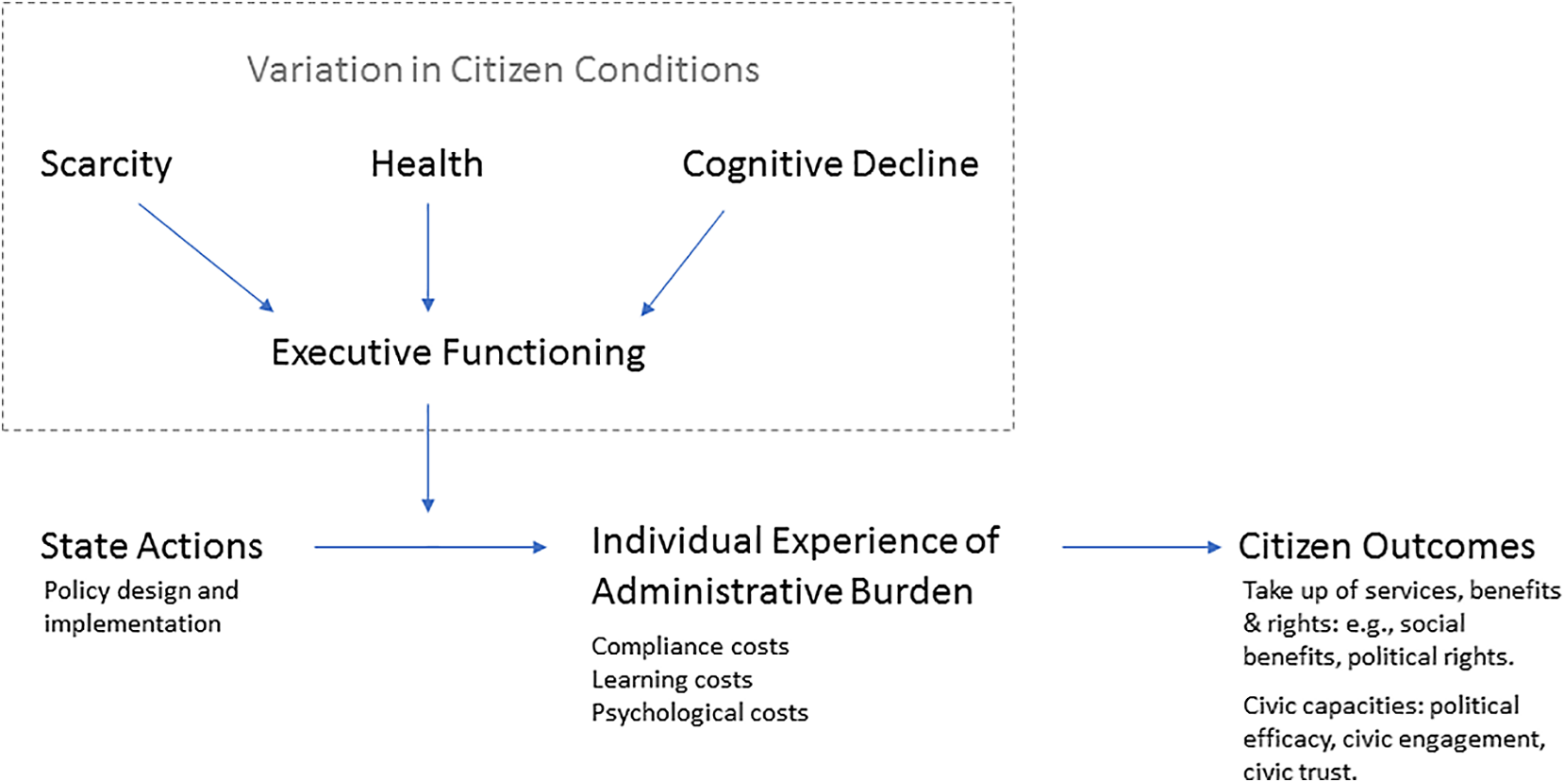
Human Capital and Administrative Burden: The Role of Executive Functioning

Defining administrative burden as the experiences of costs is helpful for two reasons. First, it distinguishes between state actions and the experiences of the individual. Figure 1—which is not intended to be a comprehensive mapping of all causal relationships—makes this distinction clear while highlighting the role of some specific types of human capital. State actions include policy design by elected officials and higher level administrative actors, as well as policy implementation practices of street‐level bureaucrats. Through such mechanisms, the state can construct rules and processes that give rise to experiences of burden. To give a simple example, the state may mandate that everyone complete a form to gain access to a benefit. That is a state action. But the form is not experienced as a burden until someone fills it out.

Second, the definition allows for individual differences in experiences of burdens and, by extension, a behavioral perspective that helps to explain those differences. For example, some may find it more difficult to complete a form, experience considerable stress because of uncertainty about the outcome, or feel stigmatized because they believe that documentation requirements signal mistrust. Others may find the form much easier to complete, feel more confident about the outcome, and not internalize compliance costs as stigmatizing. Such individual differences help explain why we observe disparities in citizen outcomes such as take‐up of services and benefits, civic engagement and feelings of political efficacy (Herd and Moynihan 2018). It thus invites efforts to explain the sources of variation in people's experiences.

Our approach is distinct from the “ordeal mechanism” perspective from economics, which proposes that people rationally weigh costs against expected benefits when engaging with the state. The implication is that the willingness to cope with burdensome state actions is a function of maximizing utility: those who wait in line, turn up for an appointment, or complete a form simply value the reward more than those unwilling to put up with the burdens. If this perspective is correct, the deliberate creation of ordeals can efficiently target benefits to the most needy (Nichols and Zeckhauser 1982). The ordeal mechanism perspective acknowledges that people may vary somewhat in their skills and resources when it comes to dealing with burdens, and ordeals might interact with factors such as the individual's health conditions (Zeckhauser 2019). But these acknowledgments serve as caveats to the model, rather than an effort to fully incorporate the role of human capital.

The actual empirical evidence on ordeal mechanisms as an efficient form of targeting is mixed (Alatas et al. 2012, 1206; Shafir 2012). In many cases, the people who would benefit most from overcoming ordeals fail to do so. For instance, take‐up of public housing benefits is especially low among the poorest citizens (Reeder 1985); challenges in managing application processes have been highlighted as one explanation for this (Currie 2006). Take‐up of the earned income tax credit is lower among eligible individuals with low incomes compared with among more well‐off citizens, and findings suggest that inabilities to overcome learning costs are part of the reason for this (Bhargava and Manoli 2015). In a study of the Temporary Assistance for Needy Families program, Brodkin and Majmundar (2010) find that beneficiaries living in deep poverty and with lower education are more likely than others to lose benefits because of failures to overcome compliance costs. Moreover, Deshpande and Li (2019) use administrative data to examine the effects of a natural experiment that increased administrative burdens: the closure of Social Security field offices, meaning that some clients have to travel further and deal with more crowded conditions to receive services. They find that closures reduce applications from eligible recipients, with the largest effects on those with moderately severe disabilities, lower education levels, and relatively low income.

Incorporating attention to the interaction of human capital and administrative burdens explains such outcomes in a way that the ordeal mechanism perspective cannot. It implies that even for programs that are targeted to specific groups, there will be variation in how people respond. In this way, variation in human capital may exacerbate inequality within groups relying on the same programs.

There is already evidence from behavioral economics about how aspects of cognition—specifically, cognitive biases—interact with burdens. People have biased risk and probability perceptions, which, in turn, affect their willingness to overcome administrative burdens. For example, people who underestimate the risks of health problems are also less likely to make the effort to overcome the hassle involved in enrolling in health insurance. Another bias affecting people's willingness to expend effort to overcome costs is being “present biased”: the tendency to overvalue the short term and hyperbolically discount long‐term outcomes (Frederick, Loewenstein, and O'Donoghue 2002). Avoiding costs in the present may therefore be preferred even if it means forgoing long‐term net benefits. The effect is that people tend to not move from their default situation. A third bias arises from choice overload or decisional conflict, which occurs when individuals feel overwhelmed by a multiplicity of choices, resulting in indecision, the selection of defaults, or poor decisions (Chernev, Böckenholt, and Goodman 2010; Jilke, Van Ryzin, and Van de Walle 2016).

Research on cognitive biases demonstrates the benefits of a behavioral approach to administrative burdens but is just the tip of the iceberg when it comes to understanding how cognition matters. In particular, this research speaks to average cognitive biases to which most people are subject, meaning that it does not inform us about individual variations in cognition and resulting responses to burdens. In the rest of this article, we theorize about how variation in one aspect of human capital—cognitive resources—leads to variation in citizens' ability to cope with state actions.

## The Role of Cognitive Resources

Before we theorize about the effects of cognitive resources on citizens' interactions with the state, it is appropriate to specify what we mean by cognitive resources. In this article, we focus on a set of cognitive functions commonly referred to as *executive functions* (Diamond 2013; Suchy 2009). While there is no universally accepted definition of executive functions (for a discussion of approaches to defining executive functions, see Suchy 2009), scholars concur that they are mental processes, mainly initiated from the prefrontal cortex of the brain, that allow humans to “engage in purposeful, goal‐directed, and future‐oriented behavior” (Suchy 2009, 109).

People with low executive functioning tend to have difficulty engaging in “deliberate thought processes such as forming goals, planning ahead, carrying out a goal‐directed plan, and performing effectively” (Dean, Schilbach, and Schofield 2017, 6). They often struggle to adapt to changing demands in their environment and to change problem‐solving approaches if current approaches are not successful. They also tend to be challenged by reduced emotional control, poor social regulation, and the inability to resist short‐sighted temptations (Beer 2012; Diamond 2013). Executive functions are particularly relevant to decision‐making and economic life (Beugré 2018; Dean, Schilbach, and Schofield 2017), and in psychological research, executive functions have been found to have a variety of behavioral and emotional effects that should, as we will argue, also affect citizens' experiences of and responses to burden in relation to interactions with the state.

Different executive functions are highly interrelated and difficult to disentangle, which is natural given the fact that they rely on the same regions in the brain. As a result, there is no consensus on how to categorize the functions. For instance, Diamond (2013) distinguishes three categories of core executive functions (inhibitory control, working memory, and cognitive flexibility) plus one higher‐order executive function (fluid intelligence). Dean, Schilbach, and Schofield (2017) distinguish three core executive functions (attention, inhibitory control, and memory) plus three higher‐order executive functions (cognitive flexibility, fluid and crystallized intelligence, and planning). We do not attempt to address these debates but instead highlight specific aspects of executive functions, where relevant, to understand the experience of burden. In the pages that follow, we discuss how executive functions may influence responses to state actions, both prior to and during citizen‐state interactions.

### *Prior to Citizen‐State Interactions*

We expect executive functions to affect eligible citizens' tendency to reach out for services and benefits from the state. For instance, executive functions may affect people's ability to manage learning costs, such as their ability to gain an overview of possibly relevant programs and services, eligibility criteria, and potential benefits. In the field of educational psychology, executive functions have been identified as essential to people's ability to learn (Barenberg, Berse, and Dutke 2011; Espy et al. 2010). The effects of executive functions on learning have been demonstrated in different educational settings, and in domains such as mathematical problem‐solving, reasoning, and language comprehension (Barenberg, Berse, and Dutke 2011, 210). And while there is certainly a difference between, for example, a student's ability to learn mathematics and an unemployed person's ability to learn about relevant state benefits, the education literature provides compelling reason to examine whether and how executive functions matter to learning costs in relation to the state.

Furthermore, executive functions may affect people's follow‐through, even if they succeed in identifying relevant programs or services. People with low executive functioning often struggle with planning and prioritizing tasks that need to be carried out, and they find it hard to delay gratification, making it difficult to get started on activities that are not pleasant. They often procrastinate, even if they want to complete certain activities (or know that they need to do so) to obtain desired long‐term rewards (Rosenblum 2012; Roth, Isquith, and Gioia 2005). Thus, citizens with low executive functioning may seek to avoid dull, time‐consuming processes or processes that give rise to psychological costs. As a result, there is reason to expect reduced participation in public services.

### *During Citizen‐State Interactions*

If citizen‐state interactions are initiated, executive functions should also be important during these interactions for a number of reasons. First, executive functions affect citizens' ability to overcome compliance costs associated with the interactions. Typically, citizens will have to comply with various kinds of requirements to receive assistance from the state. For example, people applying for the Supplemental Nutrition Assistance Program (SNAP) in the United States, informally known as food stamps, need to provide documentation of their income, meet with caseworkers, and, for many, document employment or document and participate in job training or qualifying educational activities. Similar requirements apply in relation to other public services and benefits in the United States and abroad.

Executive functions affect people's ability to plan activities ahead of time, act on those plans, and stay on task despite impulses and temptations to do something else when things get frustrating (Diamond 2013; Suchy 2009). As a result, they tend to forget about activities that need to be carried out and miss deadlines. Furthermore, when people do work on tasks, low executive functioning is associated with poorer task monitoring, meaning, for example, that people make more errors and do not catch these errors before completing the tasks (Roth, Isquith, and Gioia 2005, 22). People with low executive functioning are therefore more likely to fail to comply with compliance costs arising from conditions for receipt of public services, meaning that they will be in greater risk of sanctions and exclusion.

Second, we expect executive functions to affect citizens' psychological responses to state actions. People with low executive functioning have been found to be less psychologically resilient to major negative life events as well as more minor everyday stressors (Genet and Siemer 2011). They tend to more easily lose emotional balance and to experience excessive periods of emotional upset in reaction to frustrating circumstances (Diamond 2013). We propose that this will affect, for example, how people react to onerous rules and procedures and feelings of stigmatization while interacting with the state. Thus, lower executive functioning is likely to be associated with higher psychological costs of interacting with the state.

Third, we propose that executive functions will affect how citizens are perceived and, in turn, treated by state actors. Street‐level bureaucrats and other state actors are often motivated to help disadvantaged citizens compensate for their challenges in life (Jilke and Tummers 2018; Tummers et al. 2015). However, behaviors associated with low executive functioning (poor planning, lack of initiative, missed deadlines, etc.) can prove frustrating to state actors who may easily confuse such behaviors with lack of motivation or laziness, which may, in turn, lead them to categorize citizens as undeserving (Aarøe and Petersen 2014; Hansen 2018; Jilke and Tummers 2018). Such negative categorizations make it easier for state actors to rationalize imposing burdens and maintaining rules and requirements that they might otherwise relax for someone they perceive to be trying harder (Maynard‐Moody and Musheno 2003).

These negative dynamics may be furthered by citizens' interpersonal behavior toward and in response to the state actors. Executive functions have been shown to affect people's ability to monitor and regulate their own behavior in accordance with abstract norms of appropriateness (Beer 2012; Peterson and Welsh 2014), meaning that people with low executive functioning more often act inappropriately toward others. Failure to comply with state actors' expectations and standards of appropriate client behavior may encourage street‐level bureaucrats to ratchet up burdens and other forms of punishment (Lipsky 1980). In addition, people with low executive functioning struggle to understand other people's thoughts and actions (Diamond 2013) and to constrain their emotional and behavioral responses if they feel that others treat them badly (Denson et al. 2011). Thus, a vicious circle may emerge, encouraging state actors to not work with, or for, citizens who they find frustrating. For example, caseworkers report imposing sanctions on citizens even when they know that it offers no instrumental benefits, simply because they have grown tired of dealing with troublesome clients (Soss, Fording, and Schram 2011).

In sum, individuals with low executive functioning tend to be less able to overcome learning and compliance costs, reflected in greater difficulty in initiating and completing interactions with the state. Furthermore, these individuals may struggle to cope psychologically with state actions they perceive as demeaning and stigmatizing, and to successfully engage with state actors who perceive them as undeserving. As a consequence, citizens with low executive functioning may experience greater administrative burdens and, in turn, have a lower take‐up of relevant government benefits and services.

## The Human Capital Catch‐22: Life Factors That Create Need for Public Services and Benefits Also Impede Executive Functioning

In this section, we review evidence regarding the impact of three common life factors (experiences of scarcity, health problems, and age‐related cognitive decline) on people's executive functioning. All three factors are associated with reduced executive functioning. This basic fact has important policy implications as the poor, the sick, and the elderly constitute some of the largest social groups targeted by governmental programs. Thus, we argue that many citizens face a human capital catch‐22 in relation to interacting with the state: factors that give rise to demands for public services and benefits also impair the executive functions that, according to our theoretical arguments, are key to citizens' ability to initiate and master state interactions.

### *Scarcity and Executive Functioning*

Behavioral science, psychology, and evolutionary biology studies have shown how experiences of scarcity (sometimes referred to as harshness) affect people's cognitive resources (Frankenhuis, Panchanathan, and Nettle 2016; Mani et al. 2013; Mittal et al. 2015). Scarcity can be understood broadly as “having less [of something] than you feel you need” (Mullainathan and Shafir 2013, 4), and thus scarcity exists in many forms (e.g., financial scarcity, temporal scarcity, and loneliness or social scarcity), but the psychological effects are similar.

Evolutionary frameworks argue that because of large daily variance in foraging societies' access to resources, human psychology evolved to respond to cues of scarcity and unpredictability (Aarøe and Petersen 2013). Scarcity thereby triggers a survival mind‐set—“a relatively short‐term focus and present‐orientated attitude of taking risks” (Csathó and Birkás 2018, 2)—in which people “devalue the future and instead promote short‐term opportunism to take advantage of immediate benefits” (Mittal et al. 2015, 2).

This focusing effect of scarcity increases people's ability to meet their most immediate needs (Ariely and Wertenbroch 2002; Mullainathan and Shafir 2013, 21). However, the short‐term benefits of scarcity come at a long‐term price. Scarcity captures the mind and displaces attention from other opportunities that might also be worthy of attention (Mullainathan and Shafir 2013, 26). For instance, in lab experiments, Mani et al. (2013) told participants to imagine that they faced a large car‐repair bill and found reduced executive functioning (inhibitory control and fluid intelligence) among low‐income individuals. Indian sugarcane famers performed better in tests of executive functioning right after compared with right before harvest (Mani et al. 2013; Mullainathan and Shafir 2013). A number of studies have linked socioeconomic hardship to reduced executive functioning (inhibitory control) (e.g., Assari, Caldwell, and Mincy 2018). In effect, when people experience scarcity, they are more easily tempted to make short‐sighted decisions, such as borrowing money they cannot repay to reduce immediate financial stress (Shah, Mullainathan, and Shafir 2012).

While the research we have cited shows how scarcity shocks reduce executive functioning in the short term, other research has shown that sustained exposure to scarcity appears to also have long‐term negative effects. For example, early life experiences of scarcity have long‐lasting effects on people's executive functioning. Meta‐analyses show a medium‐size correlation between childhood socioeconomic status (SES) and executive functioning in children (Lawson, Hook, and Farah 2018) as well as executive functioning in adult life (Mittal et al. 2015, 10; Paál, Carpenter, and Nettle 2015). In the face of current economic uncertainty, adults “who grew up in lower‐SES environments were more impulsive, took more risks, and approached temptations more quickly” (Griskevicius et al. 2013, 197). In contrast, individuals who grew up in higher‐SES environments took fewer risks and displayed lower impulsivity and stronger preference for delayed rewards. Impulsivity (e.g., Fino et al. 2014; Romer et al. 2009), temporal discounting (e.g., Boyle et al. 2012), and behavioral disinhibition (e.g., Barkley 1997) are all characteristics associated with low executive functioning.

While the literature on citizen‐state interactions has not investigated the role of executive functioning directly, a number of studies have shown that people experiencing more intense poverty struggle with administrative burdens in ways consistent with our theoretical arguments and expectations (Bhargava and Manoli 2015; Brodkin and Majmundar 2010; Currie 2006; Deshpande and Li 2019).

### *Health and Executive Functioning*

Health is another aspect of human capital that can influence people's executive functioning. For example, mental health problems are often associated with reduced executive functioning. This is the case for executive function disorders, such as attention deficit hyperactivity disorder (ADHD) and attention deficit disorder (ADD) (Barkley 1997; Diamond 2005), in which low executive functions are defining symptoms. Furthermore, mental health problems such as depression (Diamond 2013; Elliott 1998), anxiety (Visu‐Petra, Miclea, and Visu‐Petra 2013), and stress (Diamond 2013; Liston, McEwen, and Casey 2009) have all been linked to a variety of executive functioning problems. It is possible that as citizens experience psychological costs in relation to interactions with the state, worsened mental health may create a negative feedback effect between experiences of burden and executive functioning, further eroding people's ability to cope with burdensome state actions.

While mental health may be the obvious means by which health matters to executive functions, physical health, and in particular the experience of physical pain, also plays a role. Like scarcity, pain tends to capture people's attention (Eccleston and Crombez 1999). This is evolutionarily beneficial as it helps people initiate actions aimed at escaping the often harmful sources of pain (e.g., removing one's hands from a hot surface), but it comes at a price in the form of reduced executive functioning (Baker et al. 2016; Eccleston and Crombez 1999; Moriarty, McGuire, and Finn 2011). The effect is not limited to short‐term pain. For instance, brain morphology studies have shown chronic pain to be associated with accelerated loss of gray matter in the prefrontal cortex, meaning that the brain region, which is considered the neural center of executive functioning, physically shrinks in response to chronic pain (Apkarian et al. 2004; Moriarty, McGuire, and Finn 2011). Medical efforts to reduce the effects of physical pain, such as opioids, may also undercut executive functioning (Moriarty, McGuire, and Finn 2011, 398).

Together with our theoretical arguments, this evidence suggests that reduced executive functioning will challenge sick people in relation to interacting with the state, which may be one explanation for empirical findings of reduced take‐up of public services and benefits among sick people. For instance, some estimates suggest that those with mental health problems are 30 percent more likely than the general public to die from cancer (Clifton et al. 2016); lower take‐up of public screening programs is one explanation for this gap (Aggarwal, Pandurangi, and Smith 2013; Clifton et al. 2016). Furthermore, studies of the Supplemental Security Income program have shown that many disabled people fail to access the program (Benitez‐Silva, Buchinsky, and Rust 2004), partly because the neediest eligible citizens struggle with the program's lengthy and complicated application process (Currie 2006), and those with severe disabilities are most affected when compliance costs increase (Deshpande and Li 2019).

### *Age‐Related Cognitive Decline and Executive Functioning*

As we grow older, our cognitive skills decline, though with significant variation in the pattern and extent of this decline across the population. In some cases, aging comes with a rapid decline associated with Alzheimer's disease or dementia, but even without those specific conditions, growing older is associated with loss of gray matter in the prefrontal cortex (Apkarian et al. 2004) and declines in fluid intelligence, which is needed for reasoning and problem‐solving. Estimates of mild cognitive impairment among adults aged 65 and older range from 10 percent to 20 percent and increase as individuals age, with the most rapid accelerations in decline and dementia happening after age 80 (Langa et al. 2008; Ritchie, Artero, and Touchon 2001).

Yet not all types of cognitive decline are equivalent in their impact. Declines in executive functioning, even more than global cognitive declines, exert the largest negative impact on older adults, threatening their ability to manage their lives and live independently (Cahn‐Weiner et al. 2000; Carlson et al. 1999; Johnson, Lui, and Yaffe 2007; Marshall et al. 2011). For example, impairments in executive functioning make it more difficult to engage in Instrumental Daily Living Tasks (IADLs), such as paying bills, doing household tasks, managing money, and following health care regimes, such as taking pharmaceuticals as prescribed (Insel et al. 2006). Many of the skills needed to manage such basic tasks are also required to engage successfully with administrative processes.

Not only do cognitive declines make it difficult to manage one's household, they also make it difficult to manage interactions with the state. Older adults struggle with access to health care and programs that provide health insurance and those experiencing cognitive decline are especially challenged. For example, older adults in the United States and elsewhere have to rely on a mix of public and private insurance options to gain comprehensive health coverage, requiring high expertise in understanding how health insurance options match with their health needs in what can be a bewildering marketplace of choices (Herd and Moynihan 2018). Less than one‐quarter of individuals enroll in the most efficient plan for their needs, resulting in an average of $300 in additional out‐of‐pocket spending each year (Heiss et al. 2013). Those with more cognitive limitations are less likely to have *any* supplemental coverage compared with those with more preserved cognitive abilities, placing the former at substantial risk of extremely high out‐of‐pocket costs (Chan and Elbel 2012). One study found that cognitive functioning was associated with knowledge about a subsidy program for low‐income Medicare beneficiaries to reduce their out‐of‐pocket health care costs (Kuye, Frank, and McWilliams 2013). Even when cognitively impaired older adults do sign up for supplemental coverage, they make poorer choices—picking plans that provide fewer benefits for higher costs (McWilliams et al. 2011).

## Discussion

The role of human capital has clear relevance for the field of public administration generally, given its historical interest in inequality. By paying attention to human capital, we can better explain the sources of those inequalities and variations in citizen‐state interactions more broadly. For example, in the study of subjective experiences of red tape (or what we label “compliance costs”), measures of human capital differences or contextual differences, such as experiences of scarcity, are likely to explain why some people find the same objective sets of rules or procedures more onerous or emotionally taxing than others (see Hattke, Hensel, and Kalucza 2019 this symposium).

We believe that important insights about distributive effects of administrative burdens can be gained by investigating empirically how reactions to burdens vary with correlates or direct indicators of human capital and cognitive functioning. This includes traditional demographic factors such as age, income, and level of education, but also indicators of respondents' physical and mental health status (well‐validated scales capturing different aspects of each exist).^1^ Studies that are able to exploit administrative data could search for indicators of, for example, financial shocks and health events. Researchers could test whether field experiments or policy‐driven natural experiments that alter administrative burdens—such as auto‐enrollment, additions of new requirements, or the provision of help with navigating state interactions—have disproportionate effects on groups with different human capital characteristics (e.g., Bhargava and Manoli 2015; Deshpande and Li 2019). Lab or survey experiments could manipulate the degree of hassle that participants face, and investigate variations in both the process by which such hassles create stress, and the outcomes that result.

The human capital catch‐22 we describe is aggravated for those cross‐pressured by multiple problems in life. For example, aging is also associated with declines in health. In the United States, about one in five older adults have a physical disability that would make it difficult for them to be mobile (Seeman et al. 2010). More than half of older adults living below 100 percent of the federal poverty level have an activity limitation, compared with 17 percent of older adults with incomes above 400 percent of the poverty level (CDC 2013). Life events such as a loss of employment does not just increase the risk of scarcity but also increases the likelihood of loneliness, depression, and other health problems that may further reduce executive functioning (Andreeva et al. 2015; Baumeister et al. 2005; Paul and Moser 2009; Yoon et al. 2017).

A large proportion of citizens will face problems like those discussed in this article over the span of their lives. This implies that human capital may provide an important mechanism for understanding the link between vulnerability to a broader range of social and environmental problems, experiences of administrative burden, and citizen outcomes such as access to public services. The emphasis on human capital offers a behavioral answer to a puzzle that confounds the ordeal mechanism account of public services: why the most needy are often less likely to take up public services, and why some struggle more than others with experiences of burdens in their interactions with the state. Low executive functioning reduces some individuals' ability to initiate and master state interactions. This, in turn, exacerbates inequalities in citizen outcomes and undermines the efficiency of government benefits and services to help the citizens for whom they were intended.

Many of our examples come from access to welfare and health benefits, but these are not the only venues in which poor, sick, and old citizens are challenged in their interactions with the state. For instance, people are less likely to vote if they have fewer financial resources (Akee et al. 2018), mental and physical health problems (Burden et al. 2017), and age‐related cognitive decline (Burden et al. 2017), which may be partly explained by reduced executive functioning.

Although we have tried to offer specific evidence on one particular aspect of human capital, executive functioning, our desire to establish depth comes at the expense of a broader exploration of the role of human capital. Even within the specific area of executive functioning, a broader range of social and environmental factors also matter, including problems of malnutrition, alcohol abuse,^2^ sleep deprivation, and noise and air pollution (Dean, Schilbach, and Schofield 2017). Beyond executive functioning, there is a great deal of room for future research to explore other aspects of human capital, such as traits or beliefs like self‐efficacy and perseverance (Bisgaard 2018) or skills such as administrative competence (Gordon 1975) or literacy (Döring 2018). Furthermore, it is important to note that lack of participation in programs can also be a function of factors other than human capital or administrative burdens.

## Conclusion

In this article, we have theorized about the importance of cognitive resources, especially executive functioning, for citizens to be able to navigate the state. We have provided illustrative examples of three common life factors—scarcity, health problems, and age‐related cognitive decline—to detail how these micro‐factors matter to interactions with the state. These factors both increase the likelihood that individuals will need assistance from the state, and undermine executive functioning skills, which may exacerbate the negative effects of burdensome interactions with the state, reducing access to state benefits and increasing inequality.

A number of practical policy implications follow from our analysis. As scholars document how burdens affect citizens, state actors have greater opportunity and obligation to consider how to balance the merits of their actions that generate burdens with the costs those burdens create. Sunstein (2019b, 1) has called for “sludge audits” to “catalogue the costs of sludge, and to decide when and how to reduce it.” Our analysis shows that such audits would be especially helpful in programs targeted toward citizens with lower executive functioning. Such at‐risk populations can be partially identified through objective criteria such as socioeconomic status, age, and mental and physical health diagnoses, but formal categories will be lower‐bound estimates because of the gap between actual incidence and formal diagnoses. For example, only a fraction of those with ADHD receive ongoing treatment for the specific condition or for mental health problems more generally (Fayyad et al. 2007).

State actions that reduce or eliminate burdens provides one mechanism to reduce the potential for variation in executive functioning generating distributive effects. For example, in the field of welfare programs, such actions include reductions in the frequency of (re)certification of eligibility, or providing a single enrollment process for multiple programs (Herd and Moynihan 2018). The technique of auto‐enrollment—in which the state uses administrative data to identify and enroll eligible individuals—provides a powerful way to level the playing field for those who struggle with administrative barriers (Herd et al. 2013).

While state actions that citizens experience as burdensome are often created for legitimate reasons—for example, to verify an applicant's eligibility in means‐tested programs—the state may seek to reduce the unintended impact such actions have by providing targeted help to those most affected. If programs cannot be designed to be simple and nudges are not enough, the provision of help—from government itself, or from third parties—offers another strategy. For example, the Affordable Care Act paid for navigators, specialists who helped new enrollees negotiate a complex health care market. Such help had a positive effect on enrollment, especially benefiting low‐income individuals, minorities, and immigrants who were more likely to be uninsured and faced more challenges with enrollment (Herd and Moynihan 2018). Underlining the political nature of not just the construction of burdens, but also their amelioration, the Donald Trump administration defunded the program. Whatever approach policy makers take, it is less and less defensible to simply ignore the implications for how the intersection of human capital and administrative burdens affect the ability of the state to provide accessible services and reduce inequality.

## Notes

1 See, e.g., the Behavior Rating Inventory of Executive Function (BRIEF‐A), which is a validated, survey‐based tool to assess executive functioning in people's everyday life (Roth, Isquith, and Gioia 2005).

2 Relatedly, in this symposium, David Weimer (2019) points to harmful addictions (including alcoholism) as an impairment to people's ability to reason about benefits they would experience relative to burdens in choice process.
